# Observation of a four-spin solid effect

**DOI:** 10.1063/5.0091663

**Published:** 2022-05-02

**Authors:** Kong Ooi Tan, Robert G. Griffin

**Affiliations:** Francis Bitter Magnet Laboratory and Department of Chemistry, Massachusetts Institute of Technology, Cambridge, Massachusetts 02139, USA

## Abstract

The two-spin solid effect (2SSE) is one of the established continuous wave dynamic nuclear polarization mechanisms that enables enhancement of nuclear magnetic resonance signals. It functions via a state-mixing mechanism that mediates the excitation of forbidden transitions in an electron–nuclear spin system. Specifically, microwave irradiation at frequencies *ω*_μ*w*_ ∼ *ω*_0S_ ± *ω*_0I_, where *ω*_0S_ and *ω*_0I_ are electron and nuclear Larmor frequencies, respectively, yields enhanced nuclear spin polarization. Following the recent rediscovery of the three-spin solid effect (3SSE) [Tan *et al.*, Sci. Adv. 5, eaax2743 (2019)], where the matching condition is given by *ω*_μ*w*_ = *ω*_0S_ ± 2*ω*_0I_, we report here the first direct observation of the four-spin solid effect (4SSE) at *ω*_μ*w*_ = *ω*_0S_ ± 3*ω*_0I_. The forbidden double- and quadruple-quantum transitions were observed in samples containing trityl radicals dispersed in a glycerol–water mixture at 0.35 T/15 MHz/9.8 GHz and 80 K. We present a derivation of the 4SSE effective Hamiltonian, matching conditions, and transition probabilities. Finally, we show that the experimental observations agree with the results from numerical simulations and analytical theory.

## INTRODUCTION

Dynamic nuclear polarization (DNP) is a technique that enhances nuclear magnetic resonance (NMR) signal intensities by transferring polarization from unpaired electrons to nearby nuclei. It requires microwave irradiation of electron–nuclear transitions and can yield a theoretical maximum enhancement factor of *ɛ* ∼658 for electron and ^1^H spin pairs.^1–3^ The excellent sensitivity bestowed by DNP results not only in a significant saving in measurement cost and time but also allows important structural information^4,5^ to be extracted from samples that were previously inaccessible. Hence, DNP has been widely applied in studying membrane proteins,^6–8^ amyloid fibrils,^9–11^ inorganic materials, and medical imaging.^2,12^ Despite the abundant successful applications, the details of the complete polarization-transfer steps and the factors affecting the DNP efficiency are still not yet fully understood. For instance, the observation of the Overhauser effect DNP in insulating solids, the attenuation of the cross effect (CE) DNP enhancements at higher fields, and the absence of a complete quantum-mechanical description involving multiple electrons in thermal mixing (TM) DNP are examples of the complexity of DNP that remains poorly understood.^13–19^

In addition to the DNP mechanisms mentioned earlier, the two-spin solid effect (2SSE) is another DNP process involving a single electron and nuclear spin. It mediates polarization transfer via excitation of forbidden electron–nuclear transitions when the microwave frequency, *ω*_μw_, is set to the sum or difference of the electron and nuclear Larmor frequencies, i.e., *ω*_0S_ ± *ω*_0I_.^20–23^ One of the reasons that the 2SSE is relatively less exploited than other DNP mechanisms is the modest DNP enhancement factor *ɛ*. A perturbation-theory-based calculation shows that the enhancement goes as ${\left(\omega_{1S}/\omega_{0I}\right)}^{2}$ and, in the last decade, ω_0I_/2*π* has increased from 200 to 800 MHz, whereas *ω*_1*S*_/2*π* has remained constant at ∼1 MHz. Thus, improved microwave sources, such as gyroamplifiers, are required to bring the 2SSE into more general usage. Furthermore, as higher Rabi fields become available, higher order solid effects could likely emerge as viable nuclear signal enhancement mechanisms.

However, at low fields, where large electron Rabi fields, *ω*_1*S*_, are currently available, we have recently reported efficient 2SSE performance with > 250 at 0.35 T with *ω*_1S_/2π ∼ 3.2 MHz using the normal SE condition (*ω*_μw_ = *ω*_0S_ ± *ω*_0I_). Additionally, the microwave field profile also shows significant peaks with *ɛ* > 100 at the *ω*_0S_ ± 2*ω*_0I_ positions, which we attributed to the three-spin solid effect (3SSE).^24^ The phenomenon was then theoretically analyzed for a three-spin system comprised of one electron and two nuclei. The analytical theory predicted that the effect is dependent on the number of 1_H_ spins surrounding the unpaired electron—a discovery that we exploited to probe the size of the spin diffusion barrier exhibited by the trityl radicals.^24–27^ The finding was recently corroborated by other approaches.^28,29^ The observation of the 3SSE prompted us to search for other higher-order transitions, and we report here our recent observation of the four-spin solid effect (4SSE), i.e., a forbidden transition present only in a four-spin system comprised of one electron and three nuclei.

**FIG. 1.**
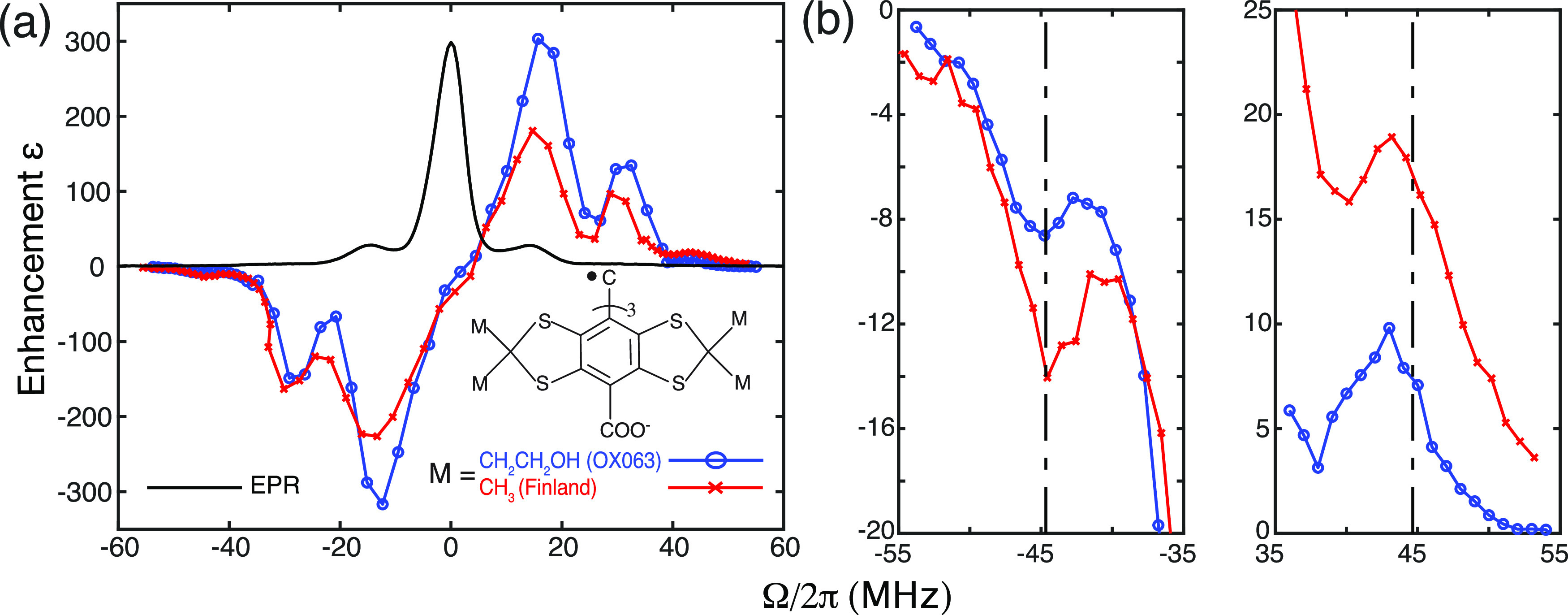
(a) The DNP field profiles of the OX063 (blue) and Finland (red) trityl DNP samples. Note that the EPR spectrum of Finland trityl (black) has an arbitrary unit on the *y*-axis, and it has a similar spectrum as trityl OX063. (b) shows an enlarged scale of the $\left|\Omega /2\pi \right|\geq 35$ MHz region shown in (a). The vertical dashed lines show the theoretically predicted 4SSE conditions at Ω = ±3*ω*_0I_.

Using a critically coupled microwave cavity and a long DNP buildup time *τ* ∼ 20 s,^24^ we observed excellent enhancement factors for the 2SSE (*ɛ* ∼300) and 3SSE (*ɛ* ∼150). Accordingly, we actively searched for the presence of the 4SSE DNP by setting the microwave offset frequencies, Ω, at three times the ^1^H Larmor frequency away from the EPR line, i.e., at Ω = *ω*_0S_ − *ω*_μ*w*_ = ±3*ω*_0I_. Figure 1 shows the full DNP field profile of trityl radicals dispersed in *d*_8_-glycerol/D_2_O/H_2_O (6:3:1 by volume) performed with a 0.35 T/9.8 GHz/15 MHz pulsed EPR/DNP spectrometer. After optimizing the microwave coupling and the NMR detection circuitry (see the “Experimental Methods” for details), we recorded DNP profiles for OX063 and Finland trityl samples. The experimental data shown in the region of $\left|\Omega /2\pi \right|\leq 35$ MHz were obtained by sweeping the microwave frequency using the ELDOR channel while maintaining the magnetic field constant, and eight scans were accumulated per data point. To ensure the optimum DNP performance, the data points in $\left|\Omega /2\pi \right|\geq 35$ MHz region were acquired by sweeping the magnetic field while keeping the microwave cavity critically coupled, and 64 scans were collected at each field position. Moreover, we used long DNP polarization times—20 s for OX063 and 33 s for Finland—to ensure that the DNP-enhanced NMR signal is saturated.

## EXPERIMENTAL METHODS

**FIG. 2.**
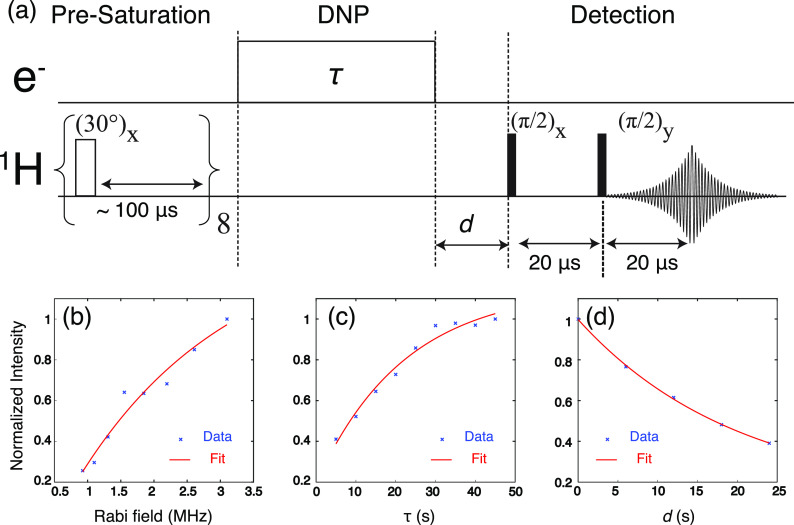
(a) Schematic diagram of DNP pulse sequence used in this work. The 4SSE performance at the DQ peak Ω/2*π* ∼ 43 MHz was measured as a function of the (b) microwave Rabi field, (c) polarization time τ, and (d) delay *d*. All experiments were performed on Finland trityl, and the data were fitted with simple single exponential curves (red) to guide the eye. The exponential rate constants obtained are 2.3 MHz, *T*_1DNP_ ∼ 22 s, and *T*_1n_ ∼ 20 s, respectively. The Rabi field was measured by performing EPR nutation experiments.

The DNP sample, instrumentation, and NMR pulse sequence [Fig. 2(a)] described here are similar to those used previously.^24^ The DNP sample is comprised of 5 mM OX063 or Finland trityl dispersed in a mixture of *d*_8_-glycerol/D_2_O/H_2_O (6:3:1 by volume), i.e., “DNP juice,” at 80 K. The experiments were performed on an X-band spectrometer (0.35 T/9.8 GHz/15 MHz for ^1^H) using an ENDOR probe (Bruker EN 4118X-MD4) with a home-built tuning-and-matching circuit. Note that it is crucial to obtain the highest *Q* value on the microwave channel via critical coupling so that the electron Rabi field can efficiently excite the strongly forbidden 4SSE transitions.^30^ We obtained an electron Rabi field of *ω*_1S_/2*π* ∼ 3 MHz using ∼10 W from a Bruker AmpX10 amplifier, and the DNP field profiles were obtained by sweeping the static *B*_0_ field while keeping the microwave irradiation *ω*_μ*w*_ frequency constant. In our original instrument arrangement, where an over coupled configuration was used, the electron Rabi field was lower (*ω*_1S_/2*π* ∼2 MHz), and we could not observe the 4SSE. Moreover, a significant improvement in NMR sensitivity and stability was achieved by terminating the EPR modulation coil in the ENDOR probe with an electrical short. This effectively minimizes the crosstalk between the NMR coil and the field modulation coil, suppressing the noise level by a factor of ∼20. Subsequently, a μ*w*-off signal could be obtained within ∼10 minutes (32 scans with a recycle delay of 20 s). We also measured the *T*_1n_ of the bulk ^1^H in the OX063 and Finland trityl DNP samples to be 26 and 22 s, respectively.

## THEORY AND NUMERICAL SIMULATIONS

We will analytically derive the effective Hamiltonian, matching condition, and transition probability of 4SSE DNP. We begin by writing down the lab-frame Hamiltonian of a four-spin e–^1^H–^1^H–^1^H system,

$$\begin{array}{ll} \hat{H} & =\Omega {\hat{S}}_{\text{z}}+\omega_{0I}\left({\hat{I}}_{1z}+{\hat{I}}_{2z}+{\hat{I}}_{3z}\right)+{\hat{S}}_{\text{z}}\left(A_{1}{\hat{I}}_{1z}+A_{2}{\hat{I}}_{2z}+A_{3}{\hat{I}}_{3z}\right)\\ & \;+{\hat{S}}_{\text{z}}\left(B_{1}{\hat{I}}_{1x}+B_{2}{\hat{I}}_{2x}+B_{3}{\hat{I}}_{3x}\right), \end{array}$$ (1)

where *ω*_0I_ = −*γB*_0_ is the nuclear Larmor frequency, *γ* is the gyromagnetic ratio (*γ*_1H_/2*π* ∼ 42.5775 MHz/T for ^1^H), and Ω is the microwave offset frequency. *A* and *B* represent the secular and pseudo-secular hyperfine coupling of the respective e–^1^H pair. We have neglected the ^1^H–^1^H dipolar couplings here because their magnitude is small (∼10 kHz) compared to the hyperfine interactions (1 MHz), and their absence simplifies the theoretical treatment. Next, we diagonalize the Hamiltonian $\hat{H}$ [Eq. (1)] to determine the energy eigenstates of the four-spin system. As the off-diagonal term $B_{1}{\hat{S}}_{\text{z}}{\hat{I}}_{\text{x}}$ does not commute with the nuclear Larmor term $\omega_{0I}{\hat{I}}_{\text{z}}$, we apply a unitary transformation on the basis set following standard procedures (see Appendixes B.2.5 of Schweiger and Jeschke^31^), i.e., we state the ansatz that $\hat{H}$ can be diagonalized by performing a unitary transformation ${\hat{H}}^{\Lambda }=\hat{U}\hat{H}{\hat{U}}^{-1}$, where $\hat{U}$ is given by

$$\begin{array}{c} \hat{U}=\exp\;\left(-i\underset{k=1,2,3}{\sum }\left(\eta_{k\alpha }{\hat{S}}_{\alpha }{\hat{I}}_{ky}+\eta_{k\beta }{\hat{S}}_{\beta }{\hat{I}}_{ky}\right)\right),\\ {\hat{S}}_{\alpha }=\frac{\hat{1}}{2}+{\hat{S}}_{\text{z}};\;\;\;{\hat{S}}_{\beta }=\frac{\hat{1}}{2}-{\hat{S}}_{\text{z}}. \end{array}$$ (2)

Then, it can be shown that the ansatz is correct if the angles *η*_*k*α/*k*β_ fulfill the following conditions:

$$\eta_{k\alpha }=\tan^{-1}\frac{-B_{k}}{A_{k}+2\omega_{0I}},\;\;\;\;\;\eta_{k\beta }=\tan^{-1}\frac{-B_{k}}{A_{k}-2\omega_{0I}},$$ (3)

where $\hat{1}$ denotes the identity operator. We implemented and expanded the diagonalization procedure to the four-spin system using Mathematica (Wolfram Research) to obtain the energy eigenvalues. Moreover, we also neglected the secular hyperfine couplings *A*_*k*_ to simplify the calculations. These terms can be safely ignored because they have negligible influence if the condition *A*_*k*_ ≪ *ω*_0I_, *ω*_1S_ is satisfied.^15^ Following that, we obtained the matching conditions for the two quantum (2Q) $\left|\alpha \beta \beta \beta^{\Lambda }\right.\;〉↔\left|\beta \alpha \alpha \alpha^{\Lambda }\right.\;〉$ and four quantum (4Q) $\left|\alpha \alpha \alpha \alpha^{\Lambda }\right.\;〉↔\left|\beta \beta \beta \beta^{\Lambda }\right.\;〉$ 4SSE transitions,

$$\begin{array}{c} \Omega =\pm \underset{k=1,2,3}{\sum }\omega_{0I}\sqrt{1+\left(B_{k}^{2}/4\omega_{0I}^{2}\right)}\\ ∼\pm 3\omega_{0I}\;if\;B_{k}\ll \omega_{0I}, \end{array}$$ (4)

and the corresponding 4Q and 2Q transition amplitudes upon microwave excitation with Rabi field, *ω*_1S_, are

$$a_{\text{i},j}=\langle \psi_{\text{i}}^{\Lambda }|\hat{U}\omega_{1S}{\hat{S}}_{\text{x}}{\hat{U}}^{-1}|\psi_{\text{j}}^{\Lambda }〉,$$ (5)

$$\begin{array}{ll} \left|a_{1,16}\right|=\left|a_{8,9}\right| & =\frac{B_{1}B_{2}B_{3}\omega_{1S}}{2\sqrt{4+\left(B_{1}^{2}/\omega_{0I}^{2}\right)}\sqrt{4+\left(B_{2}^{2}/\omega_{0I}^{2}\right)}\sqrt{4+\left(B_{3}^{2}/\omega_{0I}^{2}\right)}\omega_{0I}^{3}}\\ & \approx \frac{B_{1}B_{2}B_{3}\omega_{1S}}{16\omega_{0I}^{3}}\;if\;B\ll \omega_{0I}\;\;and\;\;\omega_{1S}\ll \omega_{0I}, \end{array}$$

where $\left|\psi^{\Lambda }\right\rangle$ are the eigenstates in ${\hat{H}}^{\Lambda }$. Next, we apply Fermi’s golden rule, which states that the transition rate between the energy eigenstates (in the case of weak perturbation) is proportional to the square of the matrix element,^22^

$$\begin{array}{c} P_{\text{DQ}}\propto {\left|a_{8,9}\right|}^{2}=\frac{B_{1}^{2}B_{2}^{2}B_{3}^{2}\omega_{1S}^{2}}{256\omega_{0I}^{6}},\\ P_{\text{QQ}}\propto {\left|a_{1,16}\right|}^{2}=\frac{B_{1}^{2}B_{2}^{2}B_{3}^{2}\omega_{1S}^{2}}{256\omega_{0I}^{6}}. \end{array}$$ (6)

Hence, the results show that the 4SSE DNP transfer efficiency has a $\omega_{0I}^{-6}$ dependence compared to the $\varepsilon \propto \omega_{0I}^{-2}$ relation exhibited by the conventional 2SSE. Besides that, Eq. (5) reveals that 4SSE depends on the product of three e–^1^H dipolar couplings, i.e., its DNP efficiency is exceptionally sensitive to the number of ^1^H spin close to the unpaired electrons.

**FIG. 3.**
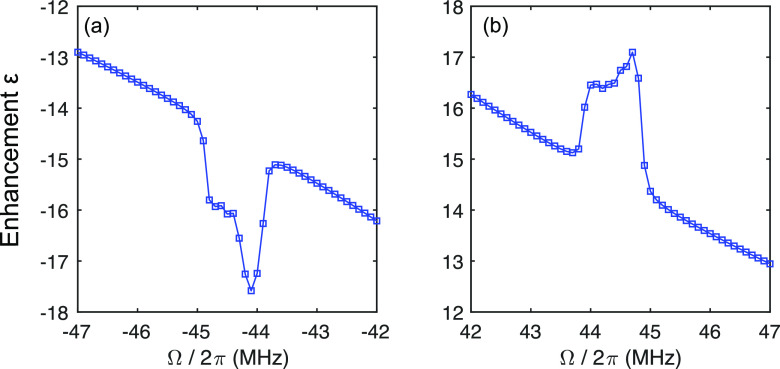
Simulated (a) quadruple-quantum (4Q) $\left|\alpha \alpha \alpha \alpha^{\Lambda }\right\rangle ↔\left|\beta \beta \beta \beta^{\Lambda }\right\rangle$ and (b) double-quantum (2Q) 2SSE $\left|\alpha \beta \beta \beta^{\Lambda }\right\rangle ↔\left|\beta \alpha \alpha \alpha^{\Lambda }\right\rangle$ DNP using the custom MATLAB program. The simulations were performed using 144 powder orientations, *γB*_1_/2*π* ∼ 4.2 MHz, *T*_1e_ ∼ 1 ms, *T*_2e_ ∼ 5 *µ*s, *T*_1n_ ∼ 13 s, *T*_2n_ ∼ 1 ms, and DNP polarization time τ ∼ 41 ms. All three e–^1^H distances were set to 4 Å, but with different sets of Euler angles in the principal-axis system (PAS), i.e., [*α*, *β*, *γ*] = [0, 0, 0]°, [5, 20, 5]°, and [10, 10, 10]°. The 4 Å distance was chosen as it represents the effective e–^1^H distance used in the literature.^24,33,34^ The electron *g*-tensor used is [2.0039, 2.0038, 2.0037] with Euler angles of [0, 40, 0]°. Note that ^1^H–^1^H couplings are not considered here.

To confirm our hypothesis that the observed DNP peaks at $\left|\Omega /2\pi \right|∼$ 45 MHz are indeed due to the 4SSE, we performed numerical simulations on a four-spin e–^1^H–^1^H–^1^H system using a customized DNP simulation package in MATLAB.^24,30,32^ Indeed, the simulated DNP field profile (Fig. 3) shows that two peaks are observed at $\left|\Omega /2\pi \right|∼$ 45 MHz. We note that the two peaks are not necessarily symmetric, a feature that was also observed in experimental results. Moreover, the widths of the simulated peaks are narrower than the experimental results [Fig. 1(b)], probably because we have not included a sufficient number of ^1^H spins and ^1^H–^1^H dipolar couplings in the simulations. Additionally, we intentionally set one of the e–^1^H hyperfine couplings to zero in the simulations and observed that the peaks at $\left|\Omega /2\pi \right|∼$ 45 MHz vanish (data not shown). This confirms that at least three ^1^H spins nearby the electron are required to yield the 4SSE.

## DISCUSSION

Figure 1(a) shows that the normal 2SSE and 3SSE peaks are very well reproduced at the Ω = ±*ω*_0I_ and Ω = ±2*ω*_0I_ positions. Moreover, the DNP enhancement on OX063 is significantly higher than our previously reported results,^24^ most likely due to a longer DNP polarization time of 20 s instead of 8 s. The microwave offset frequency is referenced to be Ω = 0 at the peak EPR intensity of the Finland trityl radical. More importantly, we observed two weak and broad peaks at microwave offset frequencies of Ω/2*π* ∼ 43 and −45 MHz [Fig. 1(b)] on both DNP samples. These peak positions are indeed very close to the predicted 4SSE peaks at Ω = ±3*ω*_0I_, where *ω*_0I_/2*π* ∼ 14.8 MHz. The peak at the 4Q position, Ω/2*π* ∼ –45 MHz, matches well with the theoretical prediction, and a slight offset of ∼1.5 MHz was observed for the 2Q peak at Ω/2*π* ∼ 43 MHz. We suspect that the origin of the small offset—relative to the width of the EPR spectrum of ∼5 MHz—could be due to the *g*-anisotropy, shift induced by higher-order Hamiltonians, or large electron–electron couplings in trityl aggregates. Although this effect is beyond the scope of this manuscript, we are planning further investigations of its origin.

We also notice that the Finland trityl has a significantly stronger 4SSE enhancement relative to OX063, despite a weaker enhancement for the 3SSE and the 2SSE conditions. This is an intriguing result given that the experiments were performed using the same experimental conditions, solvent, and spectrometer. The larger enhancement exhibited by the Finland sample at the 4SSE condition implies that the high density of nearby ^1^H nuclei (methyl groups) on the trityl molecule might be crucial in mediating the e^−^–^1^H–^1^H–^1^H SE DNP mechanism. Although the OX063 trityl molecule has more ^1^H nuclei than Finland, the methylene groups are more distant from the central carbon atom—where the unpaired electron primarily resides.^33,34^ Hence, overall electron–nuclear dipolar couplings could be smaller for OX063 and explains its weaker 4SSE performance, as suggested in Eq. (5).

To further characterize the 4SSE DNP mechanism, we measured the DNP enhancement as a function of the microwave Rabi field *γB*_1_ [Fig. 2(b)]. As expected from the low 4SSE transition probability [Eq. (5)], we observed that the DNP performance is not yet saturated despite applying *γB*_1_/2*π* ∼ 3 MHz—a field strength sufficient to saturate both the 2SSE and 3SSE DNP.^24^ Besides that, we also characterized the DNP buildup time *T*_1DNP_ of the 4SSE to be ∼22 s [Fig. 2(c)], which is only slightly longer than the *T*_1n_ ∼ 20 s [Fig. 2(d)] observed on the bulk ^1^H nuclei. These data imply that the 4SSE DNP process likely falls in the “relaxation-limited regime” where the DNP polarization could not build up further because any further polarization gain is offset by the faster ^1^H relaxation process.^35^ In such situations, what we measured is an apparent *T*_1DNP_ value that is unsurprisingly similar to the *T*_1n_ value. We expect the 4SSE DNP performance could be improved by increasing the electron Rabi field and/or lengthening the relaxation times *T*_1n_ by lowering the temperature. This will allow us to determine the non-relaxation-limited *T*_1DNP_ value that better matches the theoretically predicted transition probabilities.

## CONCLUSION

In conclusion, we have experimentally observed the 4SSE mediated by an e^−^–^1^H–^1^H–^1^H spin system. The observation is explained with an analytical theory and numerical simulations. To the best of our knowledge, this is the first report of such an effect in the DNP-NMR literature, and it illustrates that the “solid effect” consists of a family of matching conditions spaced by the nuclear Larmor frequency, *ω*_0*I*_. We note a similar four-spin mechanism reported on a system involving two electrons, a proton, and a carbon spin, i.e., a four-spin cross effect.^36^ On the other hand, Agarwal has reported recently an observation of negative cross peaks in a 2D spin diffusion MAS NMR experiment on histidine, which was attributed to a four-spin effect in a third-order Hamiltonian.^37^ As DNP/NMR technology continues to develop and improve, we expect higher sensitivity and resolution NMR spectra will become more readily available, and additional similar higher-order effects might be observed. We anticipate these findings could potentially assist in explaining anomalous experimental observations.

## Data Availability

The data that support the findings of this study are available within the article.

## References

[1] A. Abragam and M. Goldman, “Principles of dynamic nuclear polarization,” Rep. Prog. Phys. 41, 395–467 (1978). 10.1088/0034-4885/41/3/002

[2] A. S. Lilly Thankamony, J. J. Wittmann, M. Kaushik, and B. Corzilius, “Dynamic nuclear polarization for sensitivity enhancement in modern solid-state NMR,” Prog. Nucl. Magn. Reson. Spectrosc. 102–103, 120–195 (2017). 10.1016/j.pnmrs.2017.06.002 29157490

[3] T. V. Can, Q. Z. Ni, and R. G. Griffin, “Mechanisms of dynamic nuclear polarization in insulating solids,” J. Magn. Reson. 253, 23–35 (2015). 10.1016/j.jmr.2015.02.005 25797002 PMC4371145

[4] J. M. Griffiths and R. G. Griffin, “Nuclear-magnetic-resonance methods for measuring dipolar couplings in rotating solids,” Anal. Chim. Acta 283, 1081–1101 (1993). 10.1016/0003-2670(93)80267-o

[5] A. E. Bennett, R. G. Griffin, and S. Vega, “Recoupling of homo- and heteronuclear dipolar interactions in rotating solids,” NMR: Basic Princ. Prog. 33, 1–77 (1994). 10.1007/978-3-642-79127-7_1

[6] V. S. Bajaj, M. L. Mak-Jurkauskas, M. Belenky, J. Herzfeld, and R. G. Griffin, “Functional and shunt states of bacteriorhodopsin resolved by 250 GHz dynamic nuclear polarization-enhanced solid-state NMR,” Proc. Natl. Acad. Sci. U. S. A. 106, 9244–9249 (2009). 10.1073/pnas.0900908106 19474298 PMC2695048

[7] M. L. Mak-Jurkauskas, V. S. Bajaj, M. K. Hornstein, M. Belenky, R. G. Griffin, and J. Herzfeld, “Gradual winding of the bacteriorhodopsin chromophore in the first half of its ion-motive photocycle: A dynamic nuclear polarization enhanced solid state NMR study,” Proc. Natl. Acad. Sci. U. S. A. 105, 883–888 (2008). 10.1073/pnas.0706156105 18195364 PMC2242711

[8] Q. Z. Ni, T. V. Can, E. Daviso, M. Belenky, R. G. Griffin, and J. Herzfeld, “Primary transfer step in the light-driven ion pump bacteriorhodopsin: An irreversible U-turn revealed by dynamic nuclear polarization-enhanced magic angle spinning NMR,” J. Am. Chem. Soc. 140, 4085–4091 (2018). 10.1021/jacs.8b00022 29489362 PMC8313245

[9] M. J. Bayro, G. T. Debelouchina, M. T. Eddy, N. R. Birkett, C. E. MacPhee, M. Rosay, W. E. Maas, C. M. Dobson, and R. G. Griffin, “Intermolecular structure determination of amyloid fibrils with magic-angle spinning and dynamic nuclear polarization NMR,” J. Am. Chem. Soc. 133, 13967–13974 (2011). 10.1021/ja203756x 21774549 PMC3190134

[10] G. T. Debelouchina, M. J. Bayro, A. W. Fitzpatrick, V. Ladizhansky, M. T. Colvin, M. A. Caporini, C. P. Jaroniec, V. S. Bajaj, M. Rosay, C. E. Macphee, M. Vendruscolo, W. E. Maas, C. M. Dobson, and R. G. Griffin, “Higher order amyloid fibril structure by MAS NMR and DNP spectroscopy,” J. Am. Chem. Soc. 135, 19237–19247 (2013). 10.1021/ja409050a 24304221 PMC3909659

[11] S. Bahri, R. Silvers, B. Michael, K. Jaudzems, D. Lalli, G. Casano, O. Ouari, A. Lesage, G. Pintacuda, S. Linse, and R. G. Griffin, “^1^H detection and dynamic nuclear polarization–enhanced NMR of Aβ_1–42_ fibrils,” Proc. Natl. Acad. Sci. U. S. A. 119, e2114413119 (2022). 10.1073/pnas.2114413119 34969859 PMC8740738

[12] S. Jannin, J.-N. Dumez, P. Giraudeau, and D. Kurzbach, “Application and methodology of dissolution dynamic nuclear polarization in physical, chemical and biological contexts,” J. Magn. Reson. 305, 41–50 (2019). 10.1016/j.jmr.2019.06.001 31203098 PMC6616036

[13] T. V. Can, M. A. Caporini, F. Mentink-Vigier, B. Corzilius, J. J. Walish, M. Rosay, W. E. Maas, M. Baldus, S. Vega, T. M. Swager, and R. G. Griffin, “Overhauser effects in insulating solids,” J. Chem. Phys. 141, 064202 (2014). 10.1063/1.4891866 25134564 PMC4137812

[14] K. O. Tan, S. Jawla, R. J. Temkin, and R. G. Griffin, “Pulsed dynamic nuclear polarization,” eMagRes 8, 339–352 (2019).

[15] Y. Li, A. Equbal, T. Tabassum, and S. Han, “^1^H thermal mixing dynamic nuclear polarization with BDPA as polarizing agents,” J. Phys. Chem. Lett. 11, 9195–9202 (2020). 10.1021/acs.jpclett.0c01721 33058676

[16] L. Delage-Laurin, R. S. Palani, N. Golota, M. Mardini, Y. Ouyang, K. O. Tan, T. M. Swager, and R. G. Griffin, “Overhauser dynamic nuclear polarization with selectively deuterated BDPA radicals,” J. Am. Chem. Soc. 143, 20281–20290 (2021). 10.1021/jacs.1c09406 34813311 PMC8805148

[17] P. Berruyer, S. Björgvinsdóttir, A. Bertarello, G. Stevanato, Y. Rao, G. Karthikeyan, G. Casano, O. Ouari, M. Lelli, C. Reiter, F. Engelke, and L. Emsley, “Dynamic nuclear polarization enhancement of 200 at 21.15 T enabled by 65 kHz magic angle spinning,” J. Phys. Chem. Lett. 11, 8386–8391 (2020). 10.1021/acs.jpclett.0c02493 32960059

[18] A. Karabanov, G. Kwiatkowski, C. U. Perotto, D. Wiśniewski, J. McMaster, I. Lesanovsky, and W. Köckenberger, “Dynamic nuclear polarisation by thermal mixing: Quantum theory and macroscopic simulations,” Phys. Chem. Chem. Phys. 18, 30093–30104 (2016). 10.1039/c6cp04345c 27775111

[19] M. Borghini, “Spin-temperature model of nuclear dynamic polarization using free radicals,” Phys. Rev. Lett. 20, 419 (1968). 10.1103/physrevlett.20.419

[20] A. Abragam and W. G. Proctor, “Une nouvelle methode de polarization dynamique des noyaux atomiques dans les solides,” C. R. Hebdomadaries Seances Acad. Sci. 246, 2253–2256 (1958).

[21] Y. Hovav, A. Feintuch, and S. Vega, “Theoretical aspects of dynamic nuclear polarization in the solid state—The solid effect,” J. Magn. Reson. 207, 176–189 (2010). 10.1016/j.jmr.2010.10.016 21084205

[22] A. A. Smith, B. Corzilius, A. B. Barnes, T. Maly, and R. G. Griffin, “Solid effect dynamic nuclear polarization and polarization pathways,” J. Chem. Phys. 136, 015101 (2012). 10.1063/1.3670019 22239801 PMC3265031

[23] A. Henstra and W. T. Wenckebach, “Dynamic nuclear polarisation via the integrated solid effect I: Theory,” Mol. Phys. 112, 1761–1772 (2014). 10.1080/00268976.2013.861936

[24] K. O. Tan, M. Mardini, C. Yang, J. H. Ardenkjwr-Larsen, and R. G. Griffin, “Three-spin solid effect and the spin diffusion barrier in amorphous solids,” Sci. Adv. 5, eaax2743 (2019). 10.1126/sciadv.aax2743 31360772 PMC6660209

[25] W. de Boer, “Dynamic orientation of nuclei at low temperatures,” J. Low Temp. Phys. 22, 185–212 (1976). 10.1007/bf00655221

[26] M. Borghini, W. D. Boer, and K. Morimoto, “Nuclear dynamic polarization by resolved solid-state effect and thermal mixing with an electron spin-spin interaction reservoir,” Phys. Lett. A 48, 244–246 (1974). 10.1016/0375-9601(74)90487-3

[27] S. K. Jain, G. Mathies, and R. G. Griffin, “Off-resonance novel,” J. Chem. Phys. 147, 164201 (2017). 10.1063/1.5000528 29096491 PMC5659863

[28] S. K. Jain, C.-J. Yu, C. B. Wilson, T. Tabassum, D. E. Freedman, and S. Han, “Dynamic nuclear polarization with vanadium(IV) metal centers,” Chem 7, 421–435 (2021). 10.1016/j.chempr.2020.10.021

[29] Q. Stern, S. F. Cousin, F. Mentink-Vigier, A. C. Pinon, S. J. Elliott, O. Cala, and S. Jannin, “Direct observation of hyperpolarization breaking through the spin diffusion barrier,” Sci. Adv. 7, eabf5735 (2021). 10.1126/sciadv.abf5735 33931450 PMC8087418

[30] K. O. Tan, R. T. Weber, T. V. Can, and R. G. Griffin, “Adiabatic solid effect,” J. Phys. Chem. Lett. 11, 3416–3421 (2020). 10.1021/acs.jpclett.0c00654 32282219 PMC8274377

[31] A. Schweiger and G. Jeschke, Principles of Pulsed Electron Paramagnetic Resonance (Oxford University Press, 2001).

[32] K. O. Tan, C. Yang, R. T. Weber, G. Mathies, and R. G. Griffin, “Time-optimized pulsed dynamic nuclear polarization,” Sci. Adv. 5, eaav6909 (2019). 10.1126/sciadv.aav6909 30746482 PMC6357739

[33] M. K. Bowman, C. Mailer, and H. J. Halpern, “The solution conformation of triarylmethyl radicals,” J. Magn. Reson. 172, 254–267 (2005). 10.1016/j.jmr.2004.10.010 15649753

[34] S. N. Trukhan, V. F. Yudanov, V. M. Tormyshev, O. Y. Rogozhnikova, D. V. Trukhin, M. K. Bowman, M. D. Krzyaniak, H. Chen, and O. N. Martyanov, “Hyperfine interactions of narrow-line trityl radical with solvent molecules,” J. Magn. Reson. 233, 29–36 (2013). 10.1016/j.jmr.2013.04.017 23722184 PMC3713100

[35] N. A. Prisco, A. C. Pinon, L. Emsley, and B. F. Chmelka, “Scaling analyses for hyperpolarization transfer across a spin-diffusion barrier and into bulk solid media,” Phys. Chem. Chem. Phys. 23, 1006–1020 (2021). 10.1039/d0cp03195j 33404028

[36] D. Shimon, Y. Hovav, I. Kaminker, A. Feintuch, D. Goldfarb, and S. Vega, “Simultaneous DNP enhancements of ^1^H and ^13^C nuclei: Theory and experiments,” Phys. Chem. Chem. Phys. 17, 11868–11883 (2015). 10.1039/c5cp00406c 25869779

[37] V. Agarwal, “The origin of negative cross-peaks in proton-spin diffusion spectrum of fully protonated solids at fast MAS: Coherent or incoherent effect?,” J. Magn. Reson. 311, 106661 (2020). 10.1016/j.jmr.2019.106661 31869741

